# Cosmetic Surgery or Not? A Dual Perspective from Professionals and Laypeople on the Facial Aesthetic Improvement of Patients Undergoing Complex Treatments—A Pilot Study

**DOI:** 10.3390/healthcare13080947

**Published:** 2025-04-20

**Authors:** Florin Cofar, Anca Ștefania Mesaroș, Smaranda Buduru, Manuela Tăut, Ioana Gheorghiu, Tiberiu Bratu, Cosmin Sinescu

**Affiliations:** 1 Research Center in Dental Medicine Using Conventional and Alternative Technologies, Department of Prostheses Technology and Dental Materials, Faculty of Dental Medicine, “Victor Babeș” University of Medicine and Pharmacy, 9 Revolutiei 1989 Ave., 300070 Timișoara, Romania; florin.cofar@umft.ro (F.C.); sinescu.cosmin@umft.ro (C.S.); 2 Department of Dental Prosthodontics and Dental Materials, Faculty of Dentistry, “Iuliu Hațieganu” University of Medicine and Pharmacy, 32 Clinicilor Street, 400006 Cluj-Napoca, Romania; manuela.taut@elearn.umfcluj.ro; 3 Department of Medical Informatics and Biostatistics, “Iuliu Hațieganu” University of Medicine and Pharmacy, Louis Pasteur Street, Nr. 6, 400349 Cluj-Napoca, Romania; taranu.ioana@umfcluj.ro; 4 Department of Plastic and Reconstructive Surgery, Faculty of Medicine, “Victor Babeș” University of Medicine and Pharmacy, 300041 Timișoara, Romania; tiberiu.bratu@umft.ro

**Keywords:** facial aesthetics, cosmetic treatments, professional vs. layperson perception, aesthetic evaluation, treatment identification accuracy

## Abstract

**Introduction**: Facial aesthetics is an intricate domain bridging biology, psychology, and culture. It is influenced by evolutionary traits and societal norms, often driving demand for cosmetic treatments. While public perceptions of these interventions are widely studied, professional evaluations remain underexplored. This study examines differences in aesthetic judgement and treatment identification between healthcare professionals and laypeople, aiming to enhance clinical practice and research in facial aesthetics. **Methods**: A cross-sectional survey, administered via Microsoft Forms, assessed aesthetic evaluations from 88 participants, including professionals (e.g., dentists, dermatologists, and plastic surgeons) and laypeople. The survey comprised demographic questions, self-reported aesthetic assessment abilities, and visual evaluations of pre- and post-treatment images. Statistical analyses, including Fisher’s exact test and the Mann–Whitney U Test, evaluated associations between expertise level and judgments of facial beauty and treatment accuracy. **Results**: This study found no significant differences in subjective aesthetic improvement ratings between professionals and laypeople, suggesting that aesthetic judgments are inherently subjective. However, professionals demonstrated greater accuracy in identifying treatments in 3 of 7 cases (*p* < 0.05). Self-assessment revealed a critical disparity, with experts often underrating their skills compared to non-experts. **Discussion**: These findings highlight the interplay between expertise and subjectivity in aesthetic evaluations. While professional training enhances procedural recognition, biases influence judgement regardless of expertise. Incorporating standardised evaluation frameworks can refine aesthetic assessments across groups. **Conclusions**: This study underscores the value of expertise in treatment identification and advocates for standardised assessment methods to minimise bias, improve training, and support patient-centred aesthetic care.

## 1. Introduction

Facial aesthetics is a multifaceted field that intersects art, science, psychology, and culture. The human face is central to social interaction and self-identity, influencing first impressions, social standing, and personal confidence. The study of facial beauty dates back to ancient civilisations, with significant contributions from philosophers, artists, and scientists. Plato’s work on aesthetics emphasised the relationship between beauty and truth, suggesting that beauty reflects deeper truth about the object and the observer [1]. Modern studies continue to explore these philosophical insights, blending them with scientific methods to understand facial beauty.

The concept of beauty has evolved over millennia, influenced by cultural, social, and technological changes. Ancient Egyptians used intricate cosmetics and facial adornments to enhance their features, reflecting their cultural ideals of beauty. The Greeks idealised certain facial proportions, encapsulated in their art and sculpture, guided by mathematical principles like the golden ratio [2]. During the Renaissance, artists and anatomists like Leonardo da Vinci further explored these principles, studying human anatomy to achieve realistic and harmonious facial depictions [3].

Evolutionary psychology offers a compelling framework for understanding why certain facial features are universally perceived as attractive. Features such as symmetry, averageness, and sexual dimorphism are often seen as indicators of genetic fitness and reproductive potential [4]. Symmetry is associated with good health and genetic stability, while averageness suggests genetic diversity and adaptability. These preferences are thought to be evolutionarily ingrained, guiding mate selection and social interactions.

Despite universal principles, beauty standards vary significantly across cultures. Western societies often emphasise youthfulness, symmetry, and specific facial proportions as beauty ideals [5]. In contrast, East Asian cultures may prioritise fair skin, a small nose, and a V-shaped jawline [6]. These cultural differences highlight the role of social norms and media in shaping perceptions of facial beauty, underscoring the importance of a culturally sensitive approach in aesthetic studies.

Facial aesthetics has benefited from advances in medical and cosmetic interventions. Orthodontics, orthognathic surgery, and plastic surgery can create significant changes to the structure of the face, enhancing harmony and balance [7]. Non-surgical options, such as fillers and dermatological treatments, provide less invasive means of facial rejuvenation and enhancement [8]. The growing popularity of these procedures reflects an increasing desire to achieve societal beauty standards and improve self-esteem.

While the general public’s perception of facial aesthetics is well documented, less is known about how healthcare professionals view these changes. Professionals like orthodontists, plastic surgeons, and dermatologists have specialised expertise that significantly shapes their aesthetic evaluations and influences their aesthetic judgments [9]. Understanding these professional perspectives is crucial for improving patient outcomes and advancing the field of facial aesthetics.

Exploring how different groups perceive facial aesthetics has both theoretical and practical implications. For clinicians, insights into professional and patient perceptions can guide treatment planning and patient communication. For researchers, understanding the determinants of aesthetic judgments can inform future studies and improve the theoretical framework of facial aesthetics [10].

Professional expertise significantly influences aesthetic judgments. Different specialties prioritise various aspects of facial aesthetics based on their unique training and experiences [9]. For example, orthodontists focus on dental alignment and occlusion, while plastic surgeons emphasise overall facial harmony. Understanding these professional differences is essential for developing comprehensive aesthetic assessment and treatment strategies.

Ageing affects facial aesthetics by altering skin texture, volume, and structural integrity. Techniques like fat grafting, fillers, and injectable platelet-rich fibrin are used to counteract these changes and rejuvenate facial features [8]. Evaluating the effectiveness and perception of these treatments can help clinicians tailor their approaches to meet diverse patient needs and expectations.

Technological advancements have revolutionised aesthetic assessment and treatment planning. Digital imaging and simulation tools enable the precise analysis of facial features and the prediction of treatment outcomes [11]. These tools enhance the accuracy of aesthetic evaluations and improve patient satisfaction by providing clear visualisations of potential results.

Facial aesthetics significantly impact psychological well-being. Positive changes in facial appearance can enhance self-esteem, confidence, and social interactions. Conversely, dissatisfaction with one’s appearance can lead to psychological distress and social withdrawal [12]. Addressing both physical and emotional aspects of facial aesthetics is crucial for holistic patient care.

Ethical considerations are paramount in aesthetic treatments. Clinicians must balance their desire to improve aesthetics with their principles, ensuring informed consent, realistic expectations, and psychological readiness of patients [13]. Ethical practice ensures that treatments are safe, effective, and appropriate for individual circumstances.

Therefore, facial aesthetics is a complex interplay of biological, psychological, and sociocultural factors. This study aims to elucidate how different professional groups and laypersons perceive facial aesthetic changes resulting from medical and cosmetic interventions. By doing so, it seeks to contribute to facial aesthetics and provide valuable insights for clinical practice and future research.

This study aims to achieve the following:

1. Assess the self-reported abilities of different groups to judge facial beauty enhancements.
2. Evaluate perceived changes in facial aesthetics following various treatments.
3. Identify preferred treatments based on the visual assessments of before-and-after images.

## 2. Materials and Methods

### 2.1. Study Design

This study employed a cross-sectional survey design to evaluate perceptions of facial aesthetics among professionals and laypeople. The data were collected using an online questionnaire titled “Perception of Facial Aesthetics Assessment”, developed and distributed via Microsoft Forms (Appendix A). The questionnaire aimed to gather responses regarding demographic information, self-assessed ability to judge facial aesthetics, and evaluations of facial aesthetic changes based on a series of before-and-after treatment images.

### 2.2. Participants

Participants were recruited through online platforms, including social media, professional forums, and email distribution lists. The inclusion criteria were as follows:

1. Age 18 years and older.
2. Ability to provide informed consent.
3. Access to the internet to complete the online questionnaire.

The exclusion criteria were as follows:

1. Incomplete or duplicate questionnaire submissions.
2. Inconsistent responses that indicated random answering.
3. Participants who did not self-identify within the predefined professional or layperson categories.

#### Questionnaire Development

**Figure 1:**
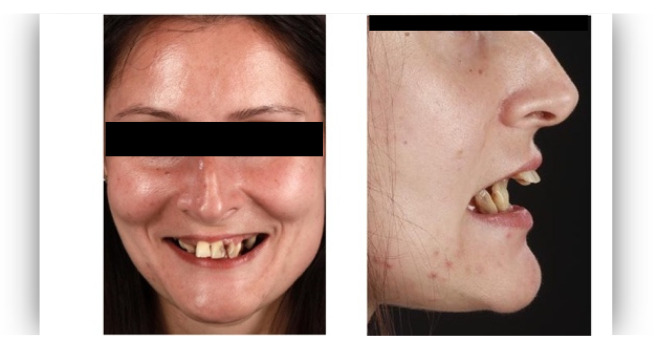
Visual assessment of the first patient before treatment in terms of the patient’s perceived age.

**Figure 2:**
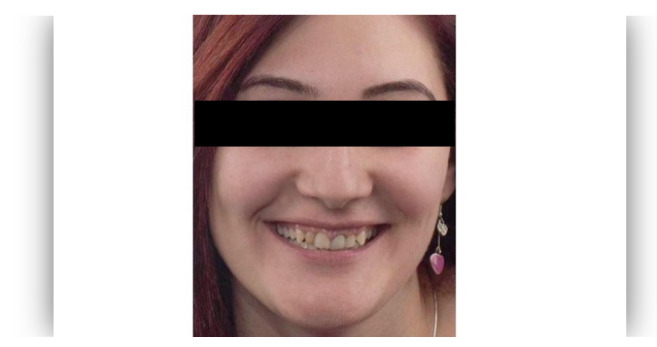
Visual assessment of the second patient before treatment in terms of the patient’s perceived age.

**Figure 3:**
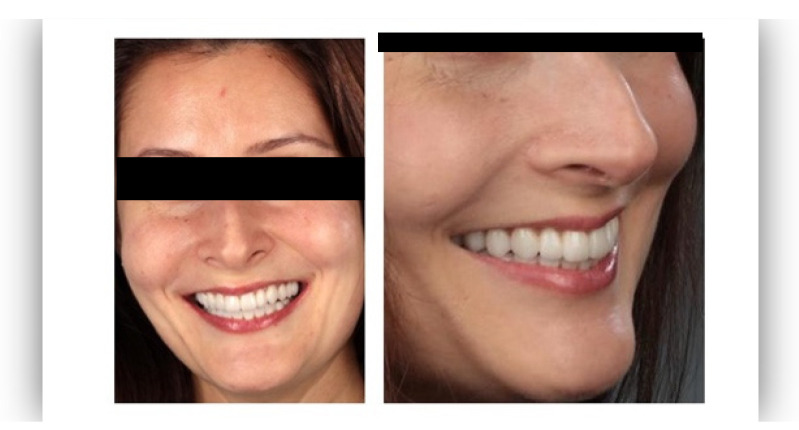
Visual assessment of the first patient after treatment in terms of the patient’s perceived age.

**Figure 4:**
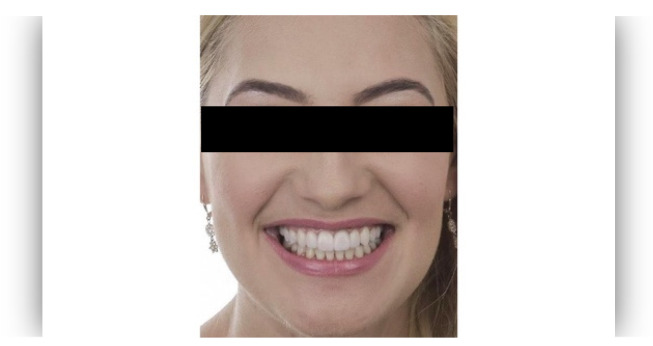
Visual assessment of the second patient after treatment in terms of the patient’s perceived age.

**Figure 5:**
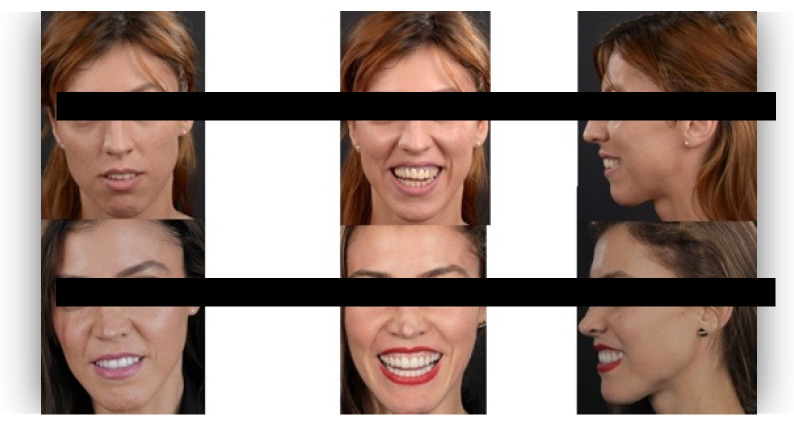
Comparative visual assessment of the first patient before and after treatment in terms of the assessment of the patient’s specific facial features together with the overall global aesthetic appearance and the evaluation of the types of treatments respondents believed the patient had undergone.

**Figure 6:**
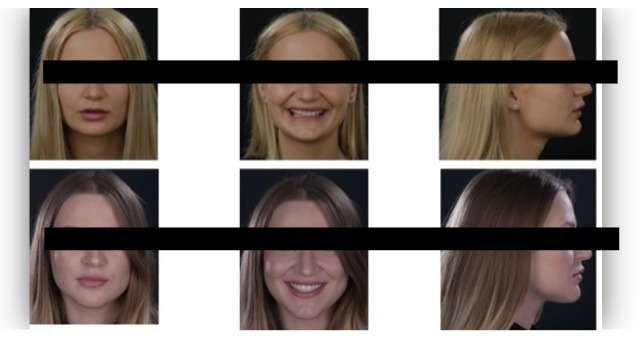
Comparative visual assessment of the second patient before and after treatment in terms of the assessment of the patient’s specific facial features together with the overall global aesthetic appearance and the evaluation of the types of treatments respondents believed the patient had undergone.

**Figure 7:**
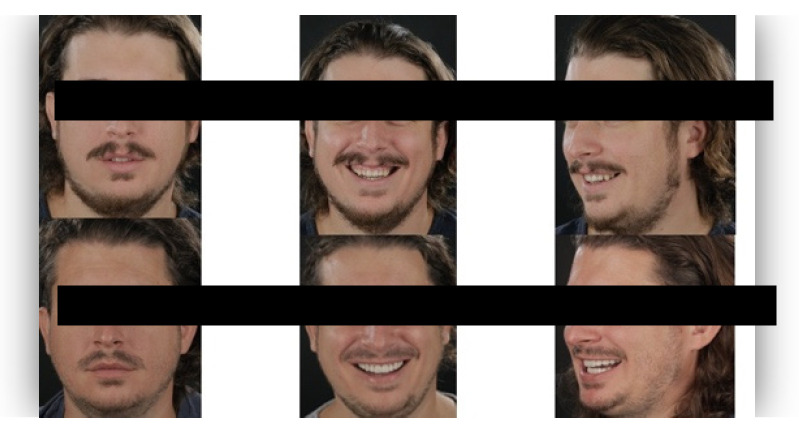
Comparative visual assessment of the third patient before and after treatment in terms of the assessment of the patient’s specific facial features together with the overall global aesthetic appearance and the evaluation of the types of treatments respondents believed the patient had undergone.

**Figure 8:**
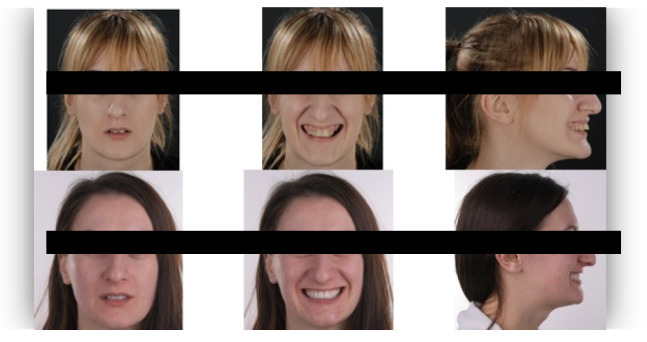
Comparative visual assessment of the fourth patient before and after treatment in terms of the assessment of the patient’s specific facial features together with the overall global aesthetic appearance and the evaluation of the types of treatments respondents believed the patient had undergone.

**Figure 9:**
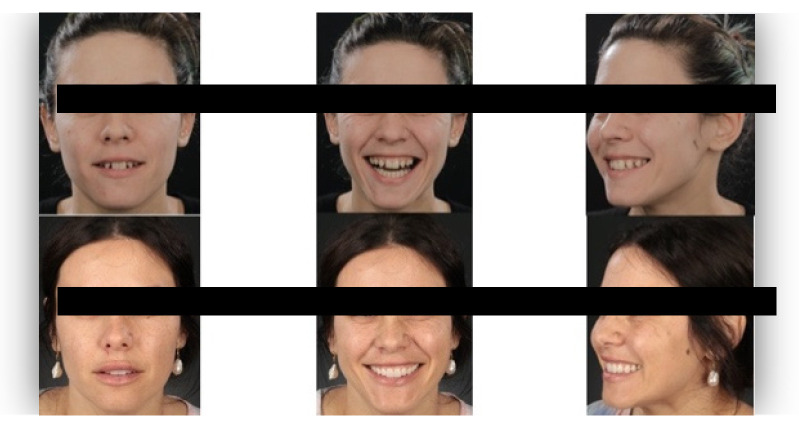
Comparative visual assessment of the fifth patient before and after treatment in terms of the assessment of the patient’s specific facial features together with the overall global aesthetic appearance and the evaluation of the types of treatments respondents believed the patient had undergone.

**Figure 10:**
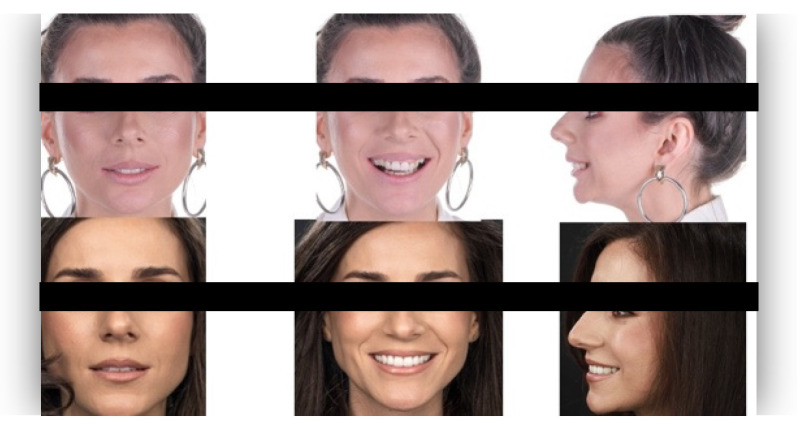
Comparative visual assessment of the sixth patient before and after treatment in terms of the assessment of the patient’s specific facial features together with the overall global aesthetic appearance and the evaluation of the types of treatments respondents believed the patient had undergone.

**Figure 11:**
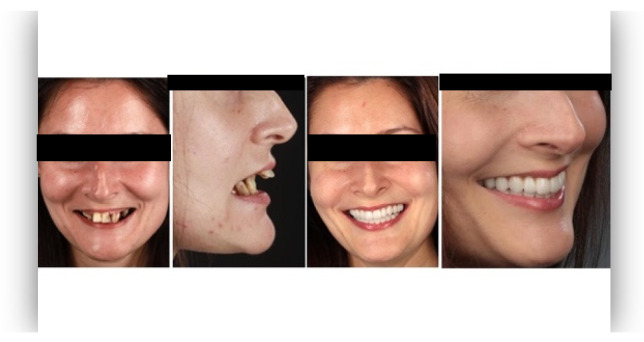
Comparative visual assessment of the seventh patient before and after treatment in terms of the assessment of the patient’s specific facial features together with the overall global aesthetic appearance and the evaluation of the types of treatments respondents believed the patient had undergone.

The questionnaire was designed to assess perceptions of facial aesthetics across several domains. It consisted of 23 questions divided into three sections:

1. Demographic Information: Questions 1 to 4 collected data on gender, residential area (urban or rural), age, and professional background.
2. Self-assessed Ability: Question 5 asked respondents to rate their ability to judge facial beauty enhancements on a scale from “basic skills” to “highly qualified”.
3. Rejuvenation Effect of Aesthetic Treatments: Questions 6–9 (the same question regarding age assessment of the depicted subject was attached to a different picture) depicted 2 patients before and after treatments (Figure 1, Figure 2, Figure 3 and Figure 4). The creators of the questionnaire recognised that the changes in patient appearance could be significant. As a result, the questions required an assessment of the patients’ ages in the initial phase and after treatment, without indicating that they were the same individuals. Figure 1 and Figure 3 depict the same patient before and after treatment, as well as Figure 2 and Figure 4. The respondents were not aware that they represented the same two patients several months apart, after undergoing treatment.
4. Assessment of Facial Aesthetic Changes: Questions 10, 12, 14, 16, 18, 20, and 22 (related to Figure 5, Figure 6, Figure 7, Figure 8, Figure 9, Figure 10 and Figure 11, respectively) presented respondents with before-and-after images of patients who had undergone various facial aesthetic treatments. Respondents were asked to evaluate changes in specific facial features and the overall aesthetic improvement using a 5-point Likert scale ranging from “significant deterioration” to “significant improvement”. At this point, the respondents were faced with “before” and “after” pictures of one subject per question, for which they had to assess modifications of special facial features.
5. Treatment Identification: Questions 11, 13, 15, 17, 19, 21, and 23, which correspond to Figure 5, Figure 6, Figure 7, Figure 8, Figure 9, Figure 10 and Figure 11, asked respondents to identify the type of treatment that they believed the patients from the previous questions had received. The response options included orthodontics, prosthodontics, orthognathic surgery, facial lifting, filler injections, dermatological therapy, and facial aesthetic surgery (such as nose and bone onlays). Again, the respondents were faced with “before” and “after” pictures of one subject per question, for which they tried to identify the treatments that the patient has undergone.

The questionnaire was constructed in a manner in which the respondents were shown one question at a time and were not able to return to previous sections, so that, even if they realised that Figure 11 was showing the same patient from Figure 1 and Figure 3, when they were shown Figure 11, they were not able to return and change their answers to questions 6 and 9 as they were in a previous section.

### 2.3. Case Description for the Seven Clinical Cases

In questions 11, 13, 15, 17, 19, 21, and 23 of the questionnaire, respondents were presented with seven distinct clinical cases (Figure 5, Figure 6, Figure 7, Figure 8, Figure 9, Figure 10 and Figure 11), each illustrating before-and-after facial images of patients who had undergone one or more facial aesthetic treatments. The objective was to evaluate participants’ ability to identify the type of treatment perceived to have been administered.

Below is a brief description of each case, including the treatments conducted for each patient:

Case 1 (Figure 5): Orthodontic treatment combined with prosthodontic rehabilitation.
Case 2 (Figure 6): Prosthodontic treatment only.
Case 3 (Figure 7): A complex interdisciplinary approach involving orthodontics, orthognathic surgery, facial aesthetic surgery (including bone onlays and/or rhinoplasty), and prosthodontics.
Case 4 (Figure 8): A combination of orthodontics and prosthodontics.
Case 5 (Figure 9): Orthodontic treatment followed by prosthodontic rehabilitation.
Case 6 (Figure 10): Orthodontic and prosthodontic treatment.
Case 7 (Figure 11): Prosthodontic treatment only.

### 2.4. Ethical Considerations

This study was conducted following the Declaration of Helsinki. Informed consent was obtained from all participants before completing the questionnaire. Participants were assured of the anonymity and confidentiality of their responses. Informed consent was also obtained from all patients presented in the figures before building the questionnaire. This study received the approval of the Ethics Commission of “Victor Babeș” University of Medicine and Pharmacy (No. Nr. 53/22 October 2024).

### 2.5. Data Collection

The online questionnaire was accessible for 5 days, from 27 October to 11 November 2024. A link to the questionnaire was distributed through various online channels, and participants were encouraged to share the link with colleagues and friends to increase the sample size. The questionnaire was designed to be completed within 10–15 min. Keeping the questionnaire concise was supposed to ensure higher response rates, as longer surveys often lead to participant fatigue and dropout. The relatively short completion time (10–15 min) was considered optimal for engagement while still allowing for meaningful data collection. This methodology was designed to maximise participation, ensure that data collection is efficient, and minimise respondent fatigue while reaching a diverse sample within a short timeframe.

The questionnaire was in the pre-testing period with a small group of professionals and laypersons (n = 8) to ensure clarity and relevance of the questions. Based on their feedback, minor adjustments were made to the wording of some questions.

### 2.6. Data Analysis

Data were exported from Microsoft Forms into Microsoft Excel for initial cleaning and organisation. Statistical analysis was performed using IBM SPSS Statistics (Version 25). Descriptive statistics were calculated for demographic variables and participants’ self-assessed ability to judge facial beauty. Mean scores and standard deviations were calculated for each facial feature to evaluate changes in facial aesthetics, including overall improvement. To compare perceptions across various professional groups and demographic categories, we applied inferential statistics, including *t*-tests, Mann–Whitney tests, and the Fisher test.

## 3. Results

### 3.1. Demographic Caractheristics and Self-Reported Level of Expertise of the Respondents

Ninety-one respondents participated in the survey, including professionals from dentistry, plastic surgery, dermatology, and cosmetology, as well as other healthcare professionals and laypersons. Three participants were excluded because they incorrectly evaluated the age of the first two female patients as between 1 and 3 years. Therefore, the final sample size was 88 respondents. The study population consisted predominantly of females, with 84.1% (74 out of 88) of the respondents female and only 15.9% (14 out of 88) male.

The distribution of respondents across various age classes is as follows: between 18 and 25 years old were 9%, between 26 and 35 years old were 28%, between 36 and 45 years old were 33%, between 46 and 55 years old were 15.73%, and between 56 and 65 years old were 14.27%. The highest representation (26–45 years: 61%) aligns with the working-age population where professionals in aesthetic fields are most active. Lower representation of younger (18–25: 9%) and older (56–65: 14.27%) groups can be explained because younger individuals may have less exposure to aesthetic treatments or professional experience while older respondents may have different aesthetic preferences or reduced engagement with online surveys.

**Figure 12:**
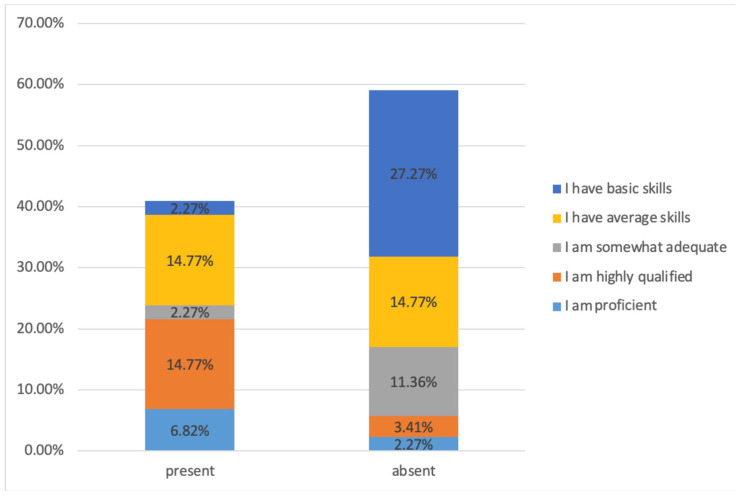
Self-reported level of expertise distribution. “present” = with expertise (dentists, dermatologists, and plastic surgeons), “absent” = without expertise (others).

Figure 12 presents the distribution of self-reported level of expertise for both categories of respondents (experts—dentists, dermatologists, plastic surgeons) and non-experts (others and laypeople).

### 3.2. Rejuvenation Effect of the Aesthetic Treatments and the Comparison of Age Evaluations by Expertise (Expert Versus Non-Expert) Before and After Treatment

**Figure 13:**
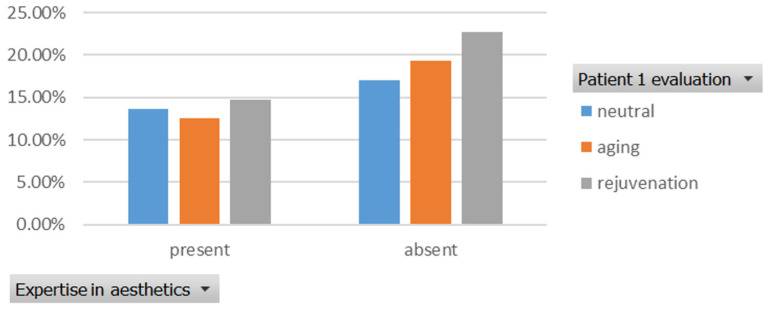
The association between the level of expertise and the evaluation of the patient’s age in the first case. “present” = with expertise (dentists, dermatologists, plastic surgeons), “absent” = without expertise (others).

The rejuvenation effect of the aesthetic treatments was assessed using the Fisher Exact Probability Test. The association between the level of expertise and the evaluation of patients’ age was tested for two cases (Figure 1, Figure 2, Figure 3 and Figure 4). For the first case, the association between the level of expertise and the evaluation of patients’ age had a *p*-value of 0.926, indicating no significant association between expertise level and age evaluation (Figure 13).

**Figure 14:**
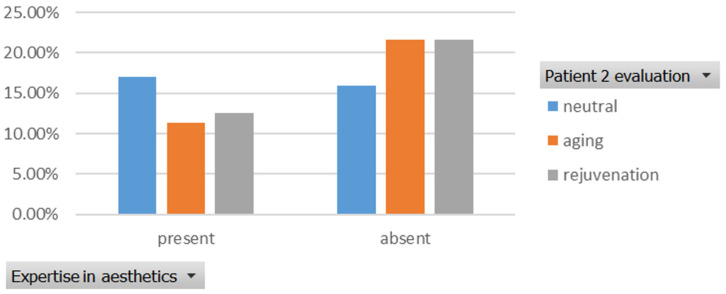
The association between the level of expertise and the evaluation of the patient’s age in the second case. “present” = with expertise (dentists, dermatologists, plastic surgeons), “absent” = without expertise (others).

Similarly, the association between the level of expertise and the evaluation of patients’ age for the second case had a *p*-value of 0.385, which also indicates no significant association between expertise level and age evaluation (Figure 14).

**Figure 15:**
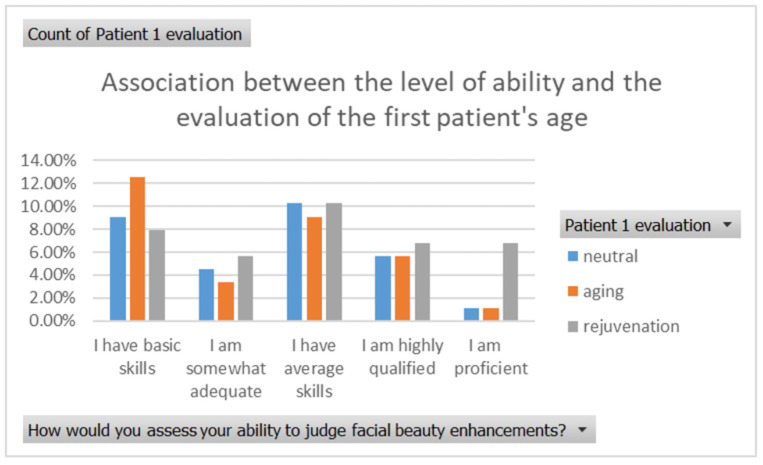
The association between self-reported level of expertise and the evaluation of the patient’s age in the first case.

The association between self-reported level of expertise and the evaluation of the patient’s age was tested for two cases (Figure 1, Figure 2, Figure 3 and Figure 4) using the Fisher Exact Probability Test. Evaluation of the first patient’s age revealed a distribution across different perceived levels of expertise. Respondents with declared basic skills had a notable proportion who judged the patient to be older than the actual age. Specifically, 12% of respondents with basic skills judged the patient to be older, while smaller percentages judged the patient to have the real age (8%) and 7% judged the patient to be younger. Among those with declared adequate skills, 4% aged the patient, 2% were neutral, and 1% observed rejuvenation. Respondents with declared average skills had a higher proportion of neutral evaluations (10%), followed by ageing (8%) and rejuvenation (5%). For those with declared high qualifications, the majority observed neutral changes (8%), with ageing and rejuvenation noted by 4% each. Declared proficient respondents mostly observed neutral changes (10%), with smaller percentages noting ageing (3%) and rejuvenation (7%) (Figure 15).

**Figure 16:**
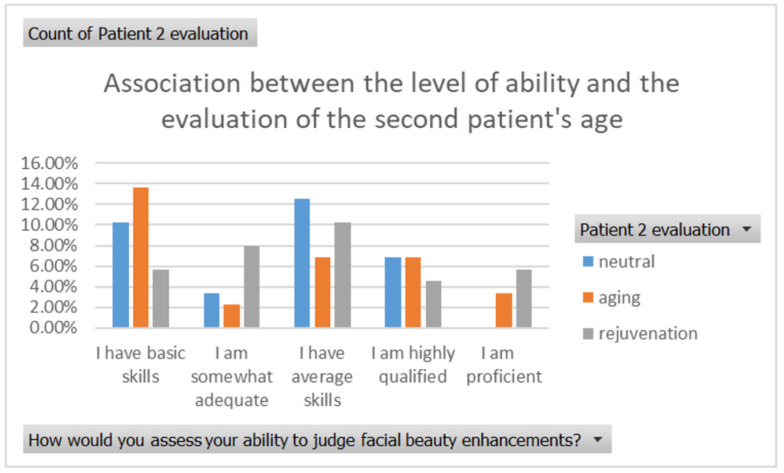
The association between self-reported level of expertise and the evaluation of the patient’s age in the second case.

The evaluation of the second patient’s age also demonstrated variability across different perceived levels of expertise. Respondents with declared basic skills had the highest proportion of ageing the patient (12%), with neutral (8%) and rejuvenation (5%) less common. Those with declared adequate skills implied ageing the patient (6%), neutral (2%), and rejuvenation (1%). Among respondents with declared average skills, neutral evaluations were most common (12%), followed by ageing (7%) and rejuvenation (4%). Declared highly qualified respondents had similar proportions for neutral (7%) and rejuvenation (6%), with ageing less noted (4%). Respondents who were deemed proficient mostly reported neutral changes (9%), with smaller proportions for ageing (2%) and rejuvenation (5%) (Figure 16).

**Figure 17:**
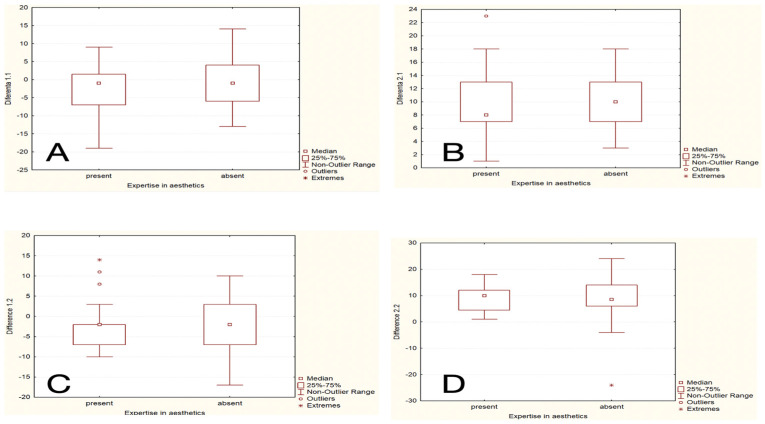
The difference between responders with versus without expertise concerning the age before and after treatment. Above and below diagrams should be compared for each of the cases. (**A**) Before treatment in the first case, (**B**) before treatment in the second case, (**C**) after treatment in the first case, and (**D**) after treatment in the second case; “present” = with expertise (dentists, dermatologists, plastic surgeons), “absent” = without expertise (others).

The differences in age evaluations by expertise (expert versus non-expert) before and after treatment were tested for the same two cases (Figure 1, Figure 2, Figure 3 and Figure 4) using the Mann–Whitney U Test. For the first patient before treatment, the Mann–Whitney U Test yielded a *p*-value of 0.585, indicating no significant difference. For the second patient before treatment, the *p*-value was 0.332, indicating no significant difference. Post-treatment evaluations showed similar results. For the first patient after treatment, the *p*-value was 0.234, indicating no significant difference between respondents with and without expertise. For the second patient after treatment, the *p*-value was 0.862, again indicating no significant difference (Figure 17A–D).

### 3.3. Overall Global Aesthetic Appearance of the Aesthetic Treatments and the Comparison in Aesthetic Evaluation Between Experts and Non-Experts

The differences in overall aesthetic evaluations by expertise (expert versus non-expert) were tested for the seven cases (Figure 5, Figure 6, Figure 7, Figure 8, Figure 9, Figure 10 and Figure 11) using the Fisher test.

**Figure 18:**
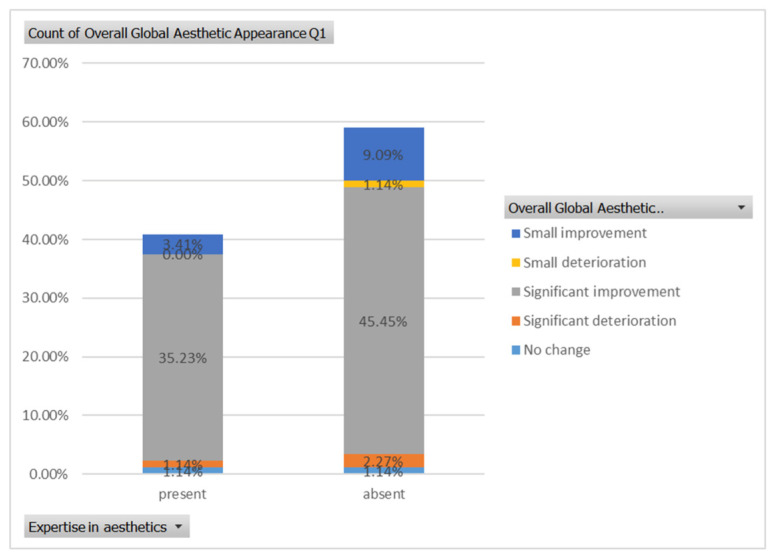
The overall aesthetic evaluations by expertise (expert versus non-expert) for the first case; “present” = with expertise (dentists, dermatologists, plastic surgeons) and “absent” = without expertise (others).

For case 1 (Figure 5), the evaluation of the overall global aesthetic appearance indicated a varied distribution of perceived changes among the respondents. A significant improvement in global aesthetic appearance was observed in 35.23% of respondents with expertise versus 45.45% of respondents without expertise (Figure 18). The aesthetic evaluation was similar between experts and non-experts (*p*-value > 0.05, the Fisher exact test).

**Figure 19:**
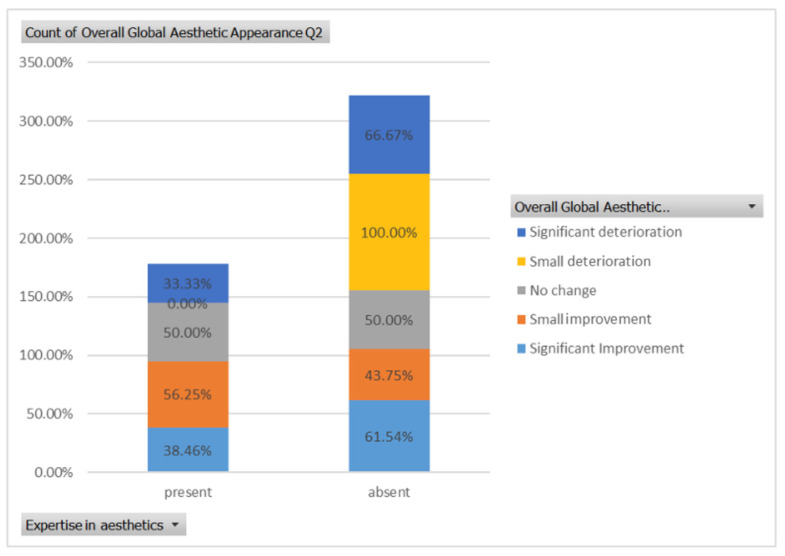
The overall aesthetic evaluations by expertise (expert versus non-expert) for the second case; “present” = with expertise (dentists, dermatologists, plastic surgeons) and “absent” = without expertise (others).

Similarly, for case 2 (Figure 6), a significant improvement in global aesthetic appearance was observed in both groups (38.46% of respondents with expertise and 61.54% of respondents without expertise). A similar percentage of experts and non-experts noticed a small improvement for this case (56.25% versus 43.75%, Figure 19). The aesthetic evaluation was similar between experts and non-experts (*p*-value > 0.05, the Fisher exact test).

**Figure 20:**
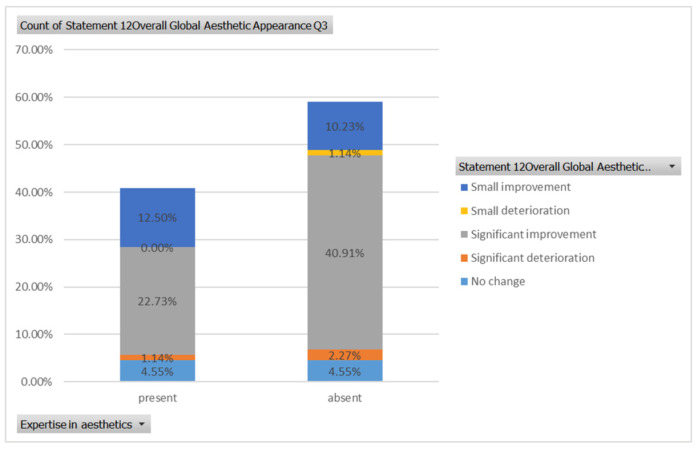
The overall aesthetic evaluations by expertise (expert versus non-expert) for the third case; “present” = with expertise (dentists, dermatologists, plastic surgeons) and “absent” = without expertise (others).

For the third case (Figure 7), a significant improvement was noticed by 22.73% of respondents with expertise versus 40.91% of respondents without expertise. A similar percentage of respondents from both groups (12.50% of experts and 10.23% of non-experts) noticed a small improvement after treatment (Figure 20). The aesthetic evaluation was similar between experts and non-experts (*p*-value > 0.05, the Fisher exact test).

**Figure 21:**
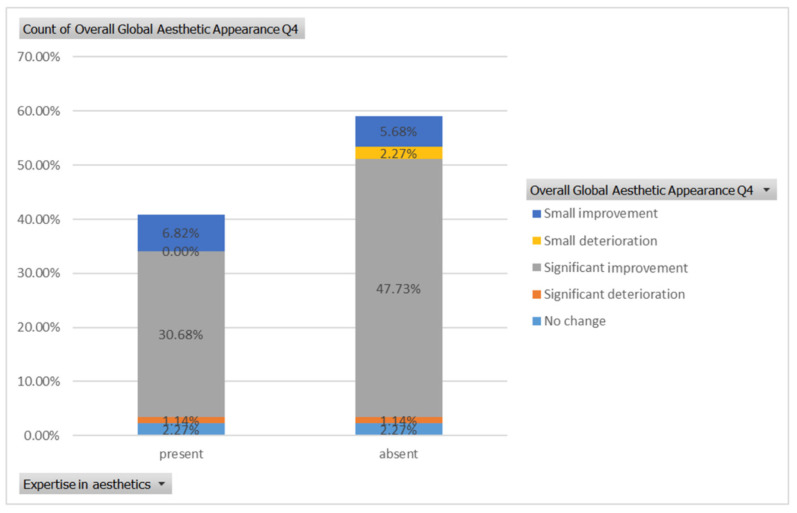
The overall aesthetic evaluations by expertise (expert versus non-expert) for the fourth case; “present” = with expertise (dentists, dermatologists, plastic surgeons), and “absent” = without expertise (others).

For the fourth case (Figure 8), a significant improvement was noticed by 30.68% of respondents with expertise versus 47.73% of respondents without expertise (Figure 21). The aesthetic evaluation was similar between experts and non-experts (*p*-value > 0.05, the Fisher exact test).

**Figure 22:**
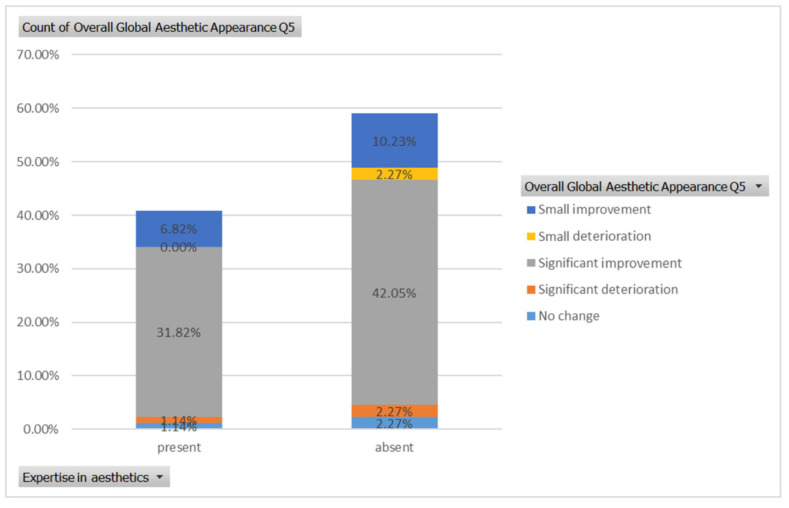
The overall aesthetic evaluations by expertise (expert versus non-expert) for the fifth case; “present” = with expertise (dentists, dermatologists, plastic surgeons) and “absent” = without expertise (others).

Similarly, a significant improvement in global aesthetic appearance was observed in both groups (31.82% of respondents with expertise and 42.05% of respondents without expertise) for case 5 (Figure 9). A small improvement in global aesthetic appearance was observed in both groups (6.82% of respondents with expertise and 2.27% of respondents without expertise, Figure 22). The aesthetic evaluation was similar between experts and non-experts (*p*-value > 0.05, the Fisher exact test).

**Figure 23:**
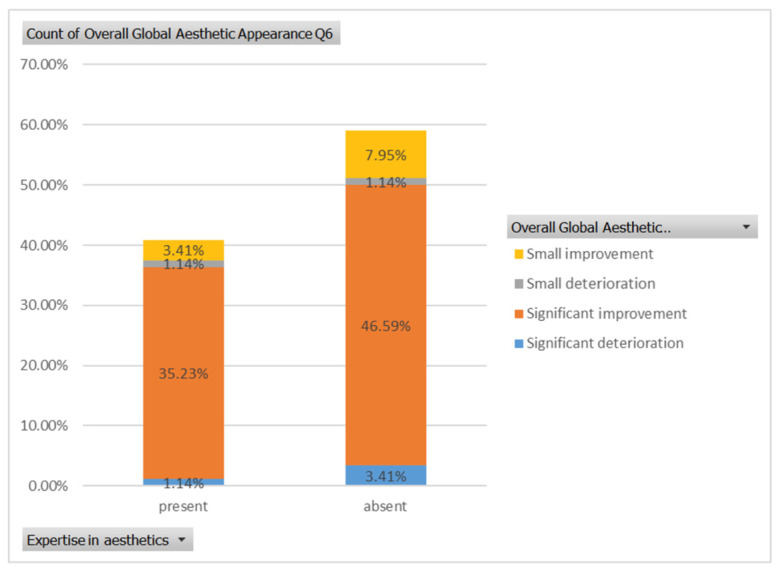
The overall aesthetic evaluations by expertise (expert versus non-expert) for the sixth case; “present” = with expertise (dentists, dermatologists, plastic surgeons) and “absent” = without expertise (others).

Regarding the sixth case (Figure 10), 35.23% of respondents with expertise in aesthetics perceived a significant improvement, while 3.41% noted a slight improvement. A small or substantial deterioration was reported by 1.14% of them in each category. In comparison, among respondents without aesthetic expertise, 46.59% indicated a significant improvement, 7.95% a slight improvement, 1.14% a small deterioration, and 3.41% a substantial decline (Figure 23). However, the aesthetic evaluation was similar between experts and non-experts (*p*-value > 0.05, the Fisher exact test).

**Figure 24:**
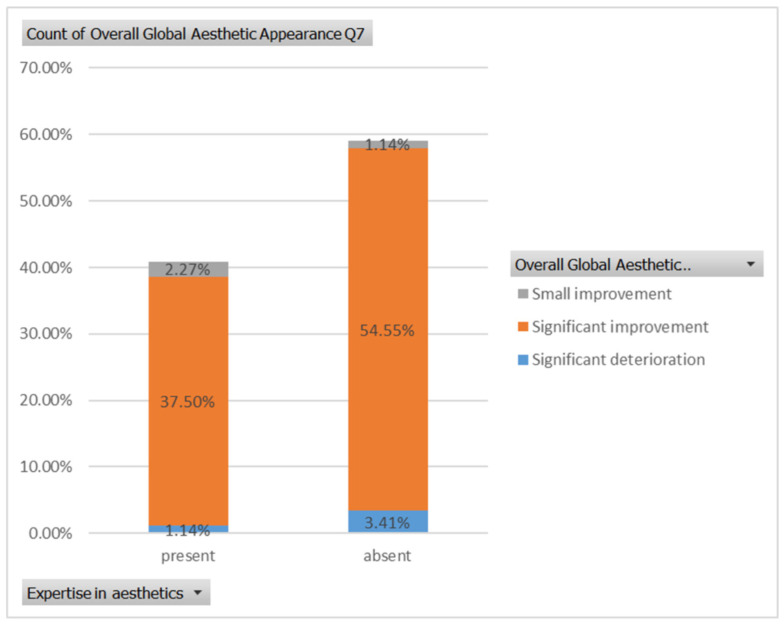
The overall aesthetic evaluations by expertise (expert versus non-expert) for the seventh case; “present” = with expertise (dentists, dermatologists, plastic surgeons) and “absent” = without expertise (others).

Finally, regarding the seventh case (Figure 11), among respondents with aesthetic expertise, 37.50% perceived a significant improvement in overall appearance, 2.27% noted a slight improvement, and 1.14% reported a significant decline. In contrast, of those without such expertise, 54.55% identified a significant improvement, 1.14% a slight improvement, and 3.41% a significant deterioration (Figure 24). The aesthetic evaluation was similar between experts and non-experts (*p*-value > 0.05, the Fisher exact test).

### 3.4. Comparison in Treatment Identification Between Experts and Non-Experts

A statistically significant association between aesthetic expertise (expert versus non-expert) and the ability to correctly assess the procedures was found for cases 1, 2, and 7 using the Mann–Whitney U Test (*p* < 0.005).

A statistically significant association between expertise in aesthetics (expert versus non-expert) and a total score consisting of the ability to estimate the dental and/or facial aesthetic procedures correctly was found for cases 1, 2, and 7 using the Mann–Whitney U Test (*p* < 0.05).

For case 1 (Figure 5), respondents with aesthetic expertise had a median total score of 87.5, with an interquartile range (IQR) from 68.75 to 100. Scores ranged from a minimum of 37.5 to a maximum of 100, with some outliers below the lower quartile. For respondents without expertise, the median total score was 62.5, with an IQR from 62.5 to 75. Scores varied from 12.5 to 100, including certain outliers that fell below the lower quartile. A statistically significant difference between the total scores of respondents with and without expertise in aesthetics was found for case 1 (*p*-value = 0.0009 using Mann–Whitney U Test).

For case 2 (Figure 6), respondents with aesthetic expertise had a median total score of 56.25, with an IQR from 37.50 to 62.50. Scores ranged from 0 to 87.50, with some outliers below the lower quartile. For respondents without expertise, the median total score was 43.75, with an IQR from 31.25 to 50.00. Scores varied from 12.50 to 75.00, with certain outliers below the lower quartile. A statistically significant difference between the total scores of respondents with and without expertise in aesthetics was found for case 2 (*p* = 0.01 using Mann–Whitney U Test).

For case 7 (Figure 11), respondents with expertise in aesthetics had a median total score of 87.5, with scores ranging from a minimum of 37.5 to a maximum of 100. For respondents without expertise, the median total score was 75.0, with scores ranging from 12.5 to 100. A statistically significant difference between the total scores of respondents with and without expertise in aesthetics was found for case 7 (*p* = 0.02 using Mann–Whitney U Test).

**Figure 25:**
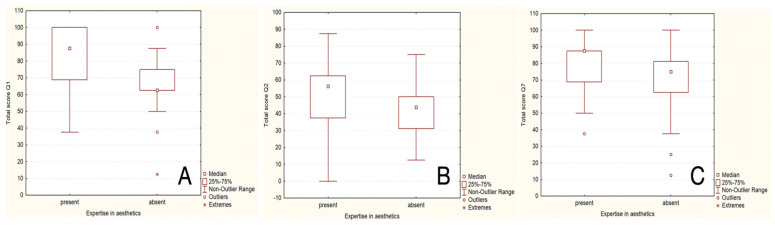
The statistically significant association between aesthetic expertise (expert versus non-expert) and the ability to correctly estimate the procedures for cases number 1, 2, and 7: (**A**) first case, (**B**) second case, and (**C**) seventh case; “present” = with expertise (dentists, dermatologists, plastic surgeons) and “absent” = without expertise (others).

Figure 25 indicates statistically significant association between aesthetic expertise (expert versus non-expert) and the ability to correctly assess the procedures for cases 1, 2, and 7.

For cases 3–6, the ability to estimate the dental and/or facial aesthetic procedures correctly was similar between experts and non-experts according to Mann–Whitney U Test (*p* > 0.05).

## 4. Discussion

This study explored how professionals and laypeople perceive facial aesthetic enhancements, providing a dual-perspective analysis. Our findings offer insight into self-perceived aesthetic expertise, subjective assessment accuracy, and the influence of professional background on treatment recognition.

First, in regard to the assessment of the self-reported abilities of different groups to judge facial beauty enhancements, the results revealed a disparity in self-reported aesthetic evaluation abilities. Experts tended to assess themselves more critically than laypeople. While 27.27% of laypeople rated their skills as “basic”, only 2.27% of experts did the same. Conversely, experts demonstrated a more critical self-assessment, with only 6.82% considering themselves “proficient”. These results align with previous studies, suggesting that professionals apply stricter self-evaluation standards, whereas laypeople often display overconfidence [14]. Demographic factors such as the predominance of female and younger respondents (aged 20–25) may have shaped these self-assessments, potentially influenced by prevailing cultural beauty standards and generational exposure to aesthetic trends. These findings underscore the need for balanced demographic representation in future studies, objective methods to assess aesthetic judgement competence and reliable aesthetic training programmes for non-experts and professionals alike.

Second, this study evaluated the perceived changes in facial aesthetics following various treatments across seven clinical cases. Statistical analysis revealed no significant differences between expert and non-expert evaluations of global aesthetic appearance. Both groups perceived treatment outcomes similarly (*p* > 0.05, Fisher’s exact test), suggesting that subjective judgments are consistent across expertise levels. These findings indicate that aesthetic evaluations are inherently subjective, supporting previous studies that emphasise the intuitive and personal nature of aesthetic perception [15]. Differences in interpretation may also depend on treatment type. In dental aesthetics, structural elements (e.g., occlusion defects or cavities) may be more easily assessed by laypeople, especially in smiling photos. In contrast, plastic surgery evaluations require a neutral facial expression and an understanding of global harmony, where expert judgement may be more decisive. These nuances highlight the importance of tailoring assessment tools to treatment context.

Two additional cases investigated changes in perceived patient age. Again, no significant associations were found between respondent expertise and age estimation accuracy. This outcome is consistent with Zaugg et al. [16], who found that subjective variables play a dominant role in age perception. These results emphasise the limitations of relying solely on professional experience for age-related assessments and advocate for the development of standardised evaluation protocols. Aesthetic training for both experts and laypeople could benefit from incorporating standardised evaluation frameworks, ensuring that aesthetic judgments remain reliable and minimising the impact of subjective variability in clinical practice. Notably, dental restoration alone can contribute to a rejuvenated appearance, even in the absence of direct facial interventions. This highlights the need for comprehensive evaluation models that consider the broader impact of dental aesthetics on overall facial perception.

Thirdly, this study evaluated the ability to identify the preferred treatments based on visual assessments of before-and-after images across seven clinical cases. The ability to visually identify treatment types was significantly higher among experts in three of the seven cases, particularly in complex procedures involving orthodontics and prosthodontics. Our findings are consistent with Milutinovic et al. [9], who concluded that professional training enhances accuracy in recognising treatment modalities and evaluating aesthetic outcomes. Such capabilities are essential not only for accurate diagnosis but also for patient communication and expectation management. Nevertheless, even among experts, subjective preferences remained a factor, underscoring the need to develop and integrate objective standards in both training and clinical assessments [17]. The study findings indicate that subjective preferences and individual judgments still play a role in aesthetic evaluations, even among experts.

Comparative studies have previously explored differences between professionals and laypeople in aesthetic judgement, especially concerning facial symmetry, dental alignment, and profile preferences. A study involving 100 Moroccan students—who exhibited no significant dentomaxillofacial dysmorphisms and had not undergone orthodontic or maxillofacial surgery—found notable discrepancies between the aesthetic assessments made by professionals and laypeople, further confirming the variability in perception between these groups [18]. According to a literature review [19], orthodontists are more sensitive to midline irregularities in dentofacial appearance than the general public due to their specialised training and experience. Minor deviations in the dental midline, often unnoticed by untrained observers, typically do not influence how attractive a smile is perceived. Another study conducted by Tauk et al. [20] highlighted differences in how orthodontists, dentists, and laypeople perceive facial profile attractiveness. The findings showed that both male and female respondents tended to prefer profiles with greater lip protrusion or orthogonal profiles, rather than those with reduced lip projection. When profiles were close to the normative range, the overall attractiveness was not necessarily influenced by the lower facial third alone. These results underscore the importance of assessing facial aesthetics by considering the entire facial profile. Overall, these findings advocate for the integration of expert evaluations in aesthetic assessments and the development of standardised measures to support objective and consistent evaluations.

Notably, the clinical cases presented in this study were selected to represent a diverse range of complex treatment scenarios as orthodontics, prosthodontics, orthognathic surgery and facial aesthetic surgery. The professional backgrounds of the respondents—dentists, dermatologists, plastic surgeons, and laypeople—significantly shaped their assessments. Experts generally provided more critical and nuanced evaluations, while laypeople tended to perceive more pronounced improvements. These differences likely stem from varying exposure to clinical outcomes and internalised aesthetic benchmarks.

To our knowledge, this is the first study in the literature to assess aesthetic evaluations of complex treatment outcomes from a dual perspective, including both professionals and laypeople. Moreover, this is the first study to evaluate simultaneously aesthetic perception, age estimation, and treatment identification using a visual assessment protocol. We believe this approach offers a novel and valuable contribution to the field.

Despite its contributions, this study has limitations. As a pilot study, it was designed primarily to explore patterns and generate hypotheses. The evaluative items used have not yet undergone formal validation. Future research should focus on expert item reviews, internal consistency testing (e.g., Cronbach’s alpha), and construct validation to ensure methodological rigour. This study also employed a non-probability convenience sampling method, which restricts the generalisability of the findings. Additionally, the variability in image quality—arising from differences in lighting, resolution, and angle due to video capture—may have affected the accuracy of aesthetic judgments. Moreover, the gender imbalance among participants, with 84.1% identifying as female represents another limitation in this study. This disproportion may be attributed to the topic and presentation of the questionnaire, which focused on facial aesthetics—a domain where the existing literature indicates greater interest and involvement among women [16,21]. While this imbalance does not undermine the internal validity of the findings, it may limit the extent to which results can be generalised across genders.

To enhance the reliability and generalisability of future studies, several improvements are recommended. First, achieving a more balanced gender distribution would help ensure that the findings reflect a wider spectrum of perspectives. Similarly, including a more diverse age range—encompassing both younger and older participants—would provide richer insights into age-related differences in aesthetic perception. Furthermore, the use of objective measures to assess participants’ expertise levels is advised, as this would reduce the subjectivity and variability associated with self-reported data. Finally, additional subgroup analyses should be conducted to determine whether the observed trends persist across different demographic segments, thereby deepening our understanding of how participant characteristics influence the outcomes.

## 5. Conclusions

Participants’ self-reported levels of expertise revealed that experts tend to assess their abilities more critically and accurately, which contributes to higher standards and promotes better self-improvement in aesthetic evaluations.

The aesthetic evaluations showed consistent results regardless of the level of expertise, indicating that aesthetic assessments can be reliable across different expertise levels when standardised criteria are used.

The consistency of the findings reinforces the need for objective and standardised methods in aesthetic evaluation, which are essential to ensure accuracy and reliability for both practitioners and patients.

Respondents with professional expertise consistently achieved higher evaluation scores, underscoring the value of specialised knowledge and training in enabling more accurate and discerning aesthetic assessments.

Finally, the findings support a more inclusive approach to training in aesthetic evaluation, emphasising that contributions from a range of professional backgrounds can enhance the overall quality and reliability of such assessments.

## Data Availability

Data are contained within this article. The data presented in this study are available upon request from the corresponding author due to ethical reasons.

## Appendix A. The Questionnaire

Perception of Facial Aesthetics Assessment

You are invited to respond to a short inquiry regarding your perception of facial aesthetics. Please give your most honest answers based on your first instinct. The inquiry does not request personal data, other than basic demographic data, and your answers are anonymous. By completing the questionnaire, you consent to the use of the data provided for scientific purposes.

1. Gender:
  ○ Male
  ○ Female
  ○ Non-binary
  ○ Prefer not to say
2. You live in:
  ○ an urban area
  ○ a rural area
3. Please select your age:
  ○ under 20
  ○ between 20–25
  ○ between 26 and 30
  ○ between 31 and 35
  ○ between 36 and 40
  ○ between 41 and 45
  ○ between 46 and 50
  ○ between 51 and 55
  ○ between 56 and 60
  ○ over 60
4. In terms of your profession, you are:
  ○ A Dentist
  ○ A Plastic Surgeon
  ○ A Dermatologist
  ○ Another kind of healthcare professional
  ○ A Cosmetologist
  ○ Other
5. How would you assess your ability to judge facial beauty enhancements:
  ○ I am proficient
  ○ I am highly qualified
  ○ I have average skills
  ○ I am somewhat adequate
  ○ I have basic skills

6–9. Please assess the age of the following patient: (Patients from Figure 1, Figure 2, Figure 3 and Figure 4)

10, 12, 14, 16, 18, 20 and 22. In the pictures below, there are the before and after treatment pictures of a patient (patients from Figure 5, Figure 6, Figure 7, Figure 8, Figure 9, Figure 10 and Figure 11). Please assess facial aesthetic changes:

	Significant deterioration	Small deterioration	No change	Small improvement	Significant improvement
Facial Profile					
Nasal Prominence					
Upper Lip Projection					
Lower Lip Projection					
Mandibular Projection					
Facial Proportions					
Paranasal Hollowing					
Lip Competence					
Facial Symmetry					
Gingival Appearance					
Labio-mental Crease					
Overall Global Aesthetic Appearance					

11, 13, 15, 17, 19, 21 and 23 What kind of treatment/s do you believe the patient has been submitted to: (patients from Figure 5, Figure 6, Figure 7, Figure 8, Figure 9, Figure 10 and Figure 11).

○ Orthodontics
○ Prosthodontics
○ Orthognathic surgery
○ Facial lifting
○ Filler injection
○ Dermatological therapy
○ Facial aesthetic surgery—nose
○ Facial aesthetic surgery—bone onlays
